# Publisher Correction: Internalized TSH receptors en route to the TGN induce local G_s_-protein signaling and gene transcription

**DOI:** 10.1038/s41467-018-07821-7

**Published:** 2018-12-19

**Authors:** Amod Godbole, Sandra Lyga, Martin J. Lohse, Davide Calebiro

**Affiliations:** 1 Institute of Pharmacology and Toxicology, University of Würzburg, Würzburg 97078, Germany; 2 Bio-Imaging Center/Rudolf Virchow Center, University of Würzburg, Würzburg, Germany; 3 Institute of Metabolism and Systems Research, University of Birmingham, Birmingham B15 2TT, UK; 4 Centre of Membrane Proteins and Receptors (COMPARE), Universities of Birmingham and Nottingham, Nottingham NG7 2RD, UK; 5 Present Address: Max Delbrück Center for Molecular Medicine, 13125 Berlin, Germany

Correction to: *Nature Communications*; 10.1038/s41467-017-00357-2; published online 5 September 2017

The original version of this Article contained errors in the three equations reported in the Methods section entitled ‘Statistics’. The incorrect forms of the equations were:

$$Y\left( t \right) = A_1\left( {1 - e^{\frac{t}{{\tau _1}}}} \right);\,t \le \Delta t_2$$

$$Y\left( t \right) = A_1\left( {1 - e^{\frac{t}{{\tau _1}}}} \right) + A_2\left( {1 - e^{\frac{{t - \Delta t_2}}{{\tau _2}}}} \right);\,\Delta t_2 < t < \Delta t_3$$

$$Y\left( t \right) = A_1\left( {1 - e^{\frac{t}{{\tau _1}}}} \right) + A_2\left( {1 - e^{\frac{{t - \Delta t_2}}{{\tau _2}}}} \right) + A_3\left( {1 - e^{\frac{{t - \Delta t_3}}{{\tau _3}}}} \right);\,t \ge \Delta t_3$$

The correct forms of the equations are:

$$Y\left( t \right) = A_1\left( {1 - e^{ - \frac{t}{{\tau _1}}}} \right);\,t \le \Delta t_2$$

$$Y\left( t \right) = A_1\left( {1 - e^{ - \frac{t}{{\tau _1}}}} \right) + A_2\left( {1 - e^{ - \frac{{t - \Delta t_2}}{{\tau _2}}}} \right);\,\Delta t_2 < t < \Delta t_3$$

$$Y\left( t \right) = A_1\left( {1 - e^{ - \frac{t}{{\tau _1}}}} \right) + A_2\left( {1 - e^{ - \frac{{t - \Delta t_2}}{{\tau _2}}}} \right) + A_3\left( {1 - e^{ - \frac{{t - \Delta t_3}}{{\tau _3}}}} \right);\,t \ge \Delta t_3$$

These errors have been corrected in both the PDF and HTML versions of the article.

