# Supplementary material for: Internalized TSH receptors en route to the TGN induce local Gs-protein signaling and gene transcription
Source: Nat Commun. 2017 Sep 5;8:443. doi: 10.1038/s41467-017-00357-2 (PMC5585343; doi:10.1038/s41467-017-00357-2)
Supplement: Supplementary file 1 — Supplementary Information [file 41467_2017_357_MOESM1_ESM.pdf]

File Name: Supplementary Information
Descriptions: Supplementary Figures and Supplementary Table

File Name: Peer Review File
Descriptions:

File Name: Supplementary Movie 1
Descriptions: Progressive accumulation of internalized TSH/TSH receptor complexes in the TGN. The movie shows the image sequence of Figure 1c after removal of the fluorescent ligand. Frames were acquired every 5 s. Playback is accelerated (10 frames/s).

File Name: Supplementary Movie 2
Descriptions: Zoomed-in movie showing the fusion of vesicles carrying TSH/TSH receptor complexes with the TGN. The movie corresponds to the inset in Figure 1e. Frames were acquired every 5 s. Playback is accelerated (10 frames/s).

File Name: Supplementary Movie 3
Descriptions: Dynamics of asymmetrical structure containing TSH/TSH receptor complexes and Gs-protein. Shown is a representative structure filmed in a primary mouse thyroid cell that was transfected with Gs-YFP (green) and stimulated with TSH-594 (magenta). Frames were acquired every 3 s. Playback is accelerated (10 frames/s).

File Name: Supplementary Movie 4
Descriptions: Arrival of new TSH containing vesicle leads to enhancement of Nb37-YFP signal. Shown are two consecutive representative events during which the Nb37-YFP signal (orange) is enhanced upon arrival of new TSH-containing vesicles (magenta). The movie corresponds to the time series shown in Figure 3c. Frames were acquired every 5 s. Playback is accelerated (5 frames/s).

Supplementary Figures

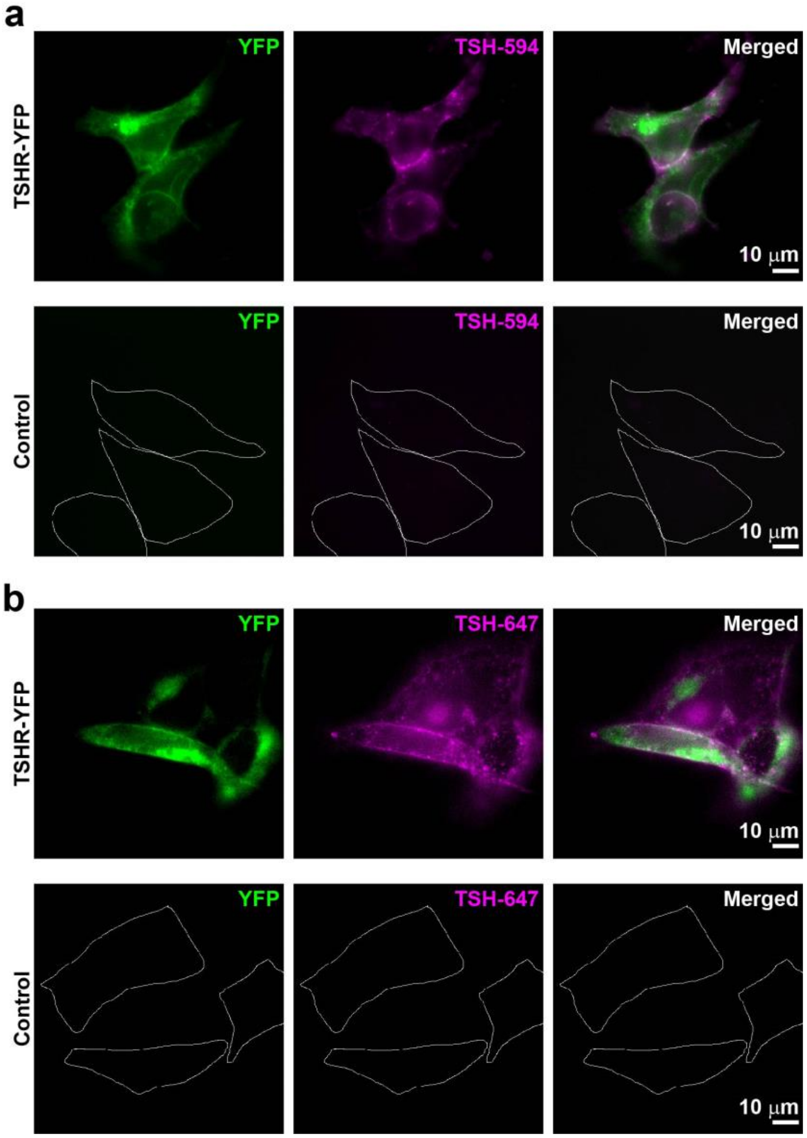

Supplementary Figure 1: Fluorescent TSH binds specifically to the TSH receptor expressed in HEK293 cells. Human embryonic kidney (HEK293) cells were transfected with TSHR-YFP or the empty expression vector (control) and stimulated with TSH-594 (**a**) or TSH-647 (**b**) for 10 min. Shown are epifluorescence images at the end of the stimulation. Note the TSH bound to the plasma membrane only in TSHR-YFP transfected cells. Cell edges in control are marked in white. Data are representative of 3 independent experiments.

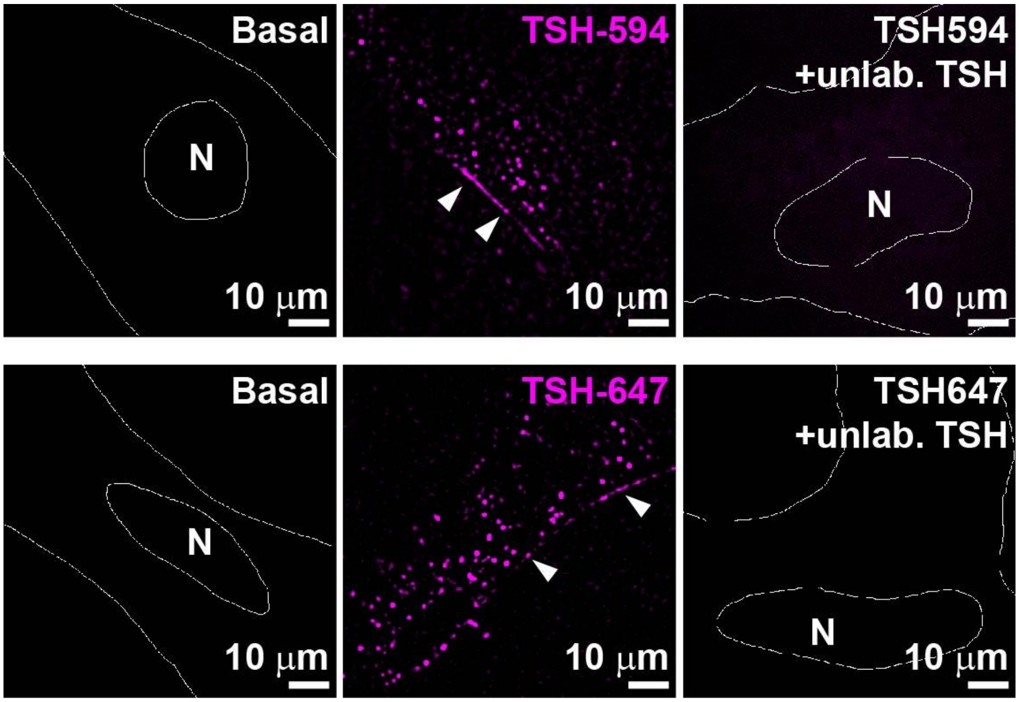

Supplementary Figure 2: Fluorescent TSH binds specifically to primary mouse thyroid cells. Primary mouse thyroid cells were stimulated with fluorescent TSH alone or with fluorescent TSH in the presence of a 100-fold molar excess of unlabeled TSH to competitively inhibit binding of fluorescent TSH. Where appropriate, cell edges and nucleus (N) are marked in white. Data are representative of 3 independent experiments.

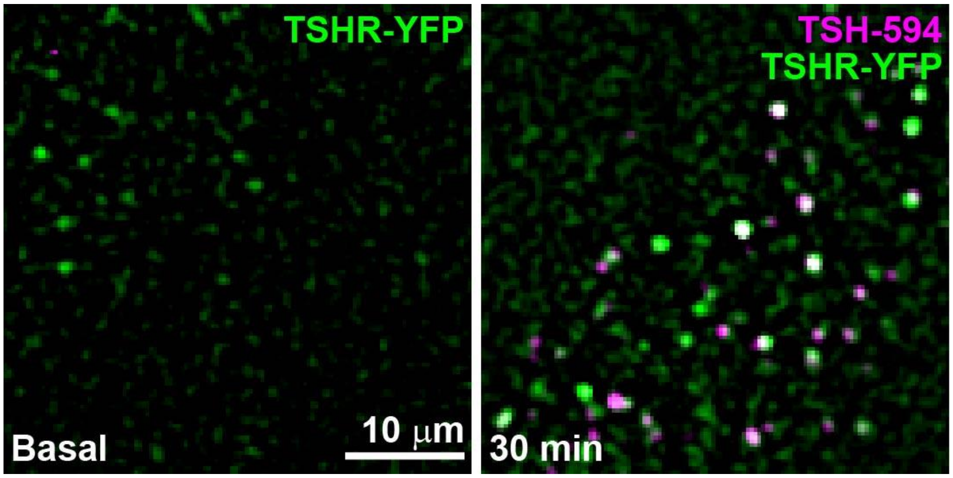

Supplementary Figure 3: Simultaneous visualization of TSH and TSH receptors. Primary mouse thyroid cells transfected with TSHR-YFP (green) were stimulated with TSH-594 (magenta) for 10 min. Representative images of the same cell obtained before (basal) and 30 min from beginning of stimulation are shown. White indicates colocalization.

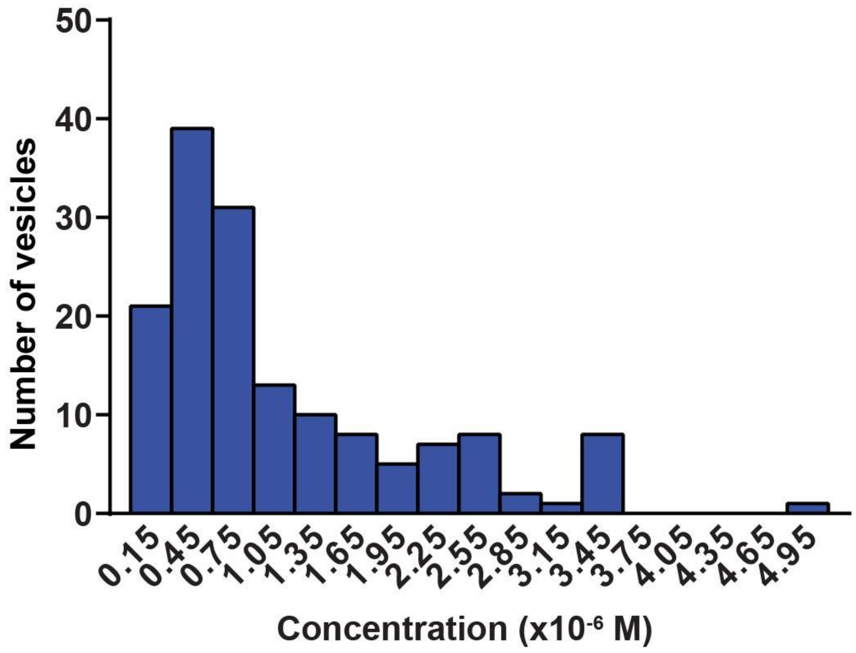

Supplementary Figure 4: Histogram showing the estimated concertation of internalized fluorescent TSH within intracellular vesicular structures. Values were obtained based on a single-molecule calibration with fluorescent TSH as explained in Methods.

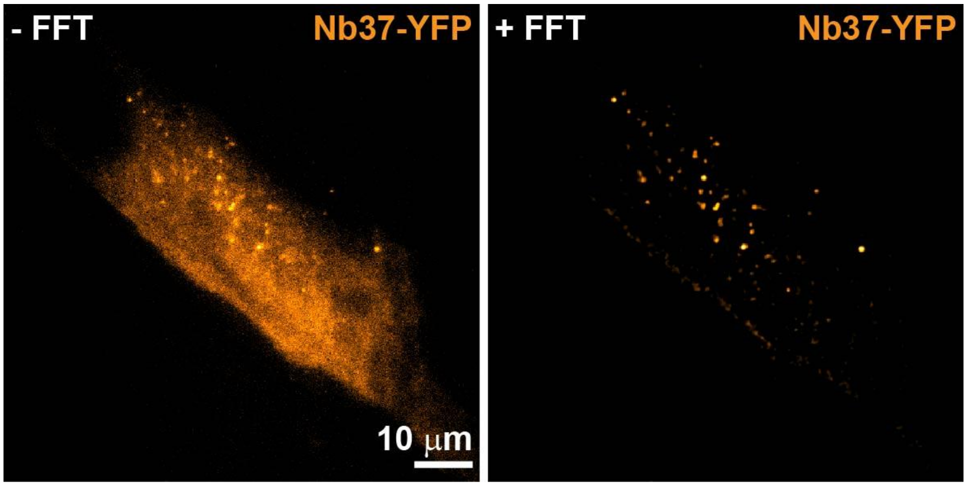

Supplementary Figure 5: Processing of Nb37-YFP images. Left panel, representative raw image of a primary mouse thyroid cells transfected with nanobody Nb37-YFP (orange). Nb37-YFP was present in the cytosol as well as on intracellular membranes. Right panel, same image after application of a Fast Fourier Transform (FFT) bandpass filter to suppress the cytosolic signal for better visualization.

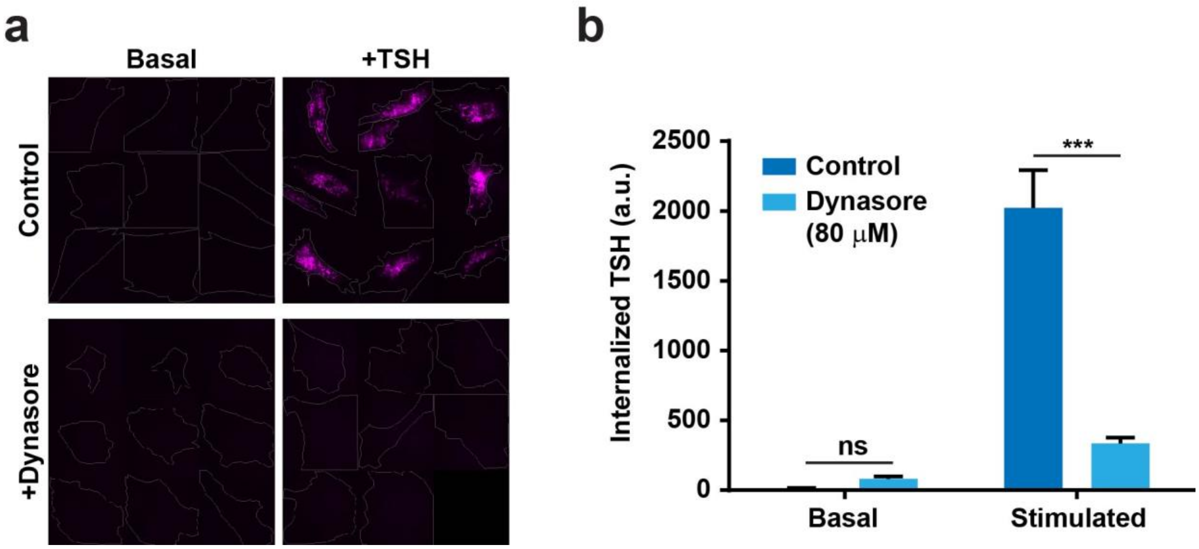

Supplementary Figure 6: Efficacy of dynasore in inhibiting TSH/TSH receptor internalization. Primary mouse thyroid cells were stimulated with TSH-594 for 10 min with or without (control) preincubation with dynasore (80 μM) **(a)** Montages showing representative HILO images of cells in each condition obtained 30 min after begin of stimulation. TSH-594 is not visible on the plasma membrane of control cells as it was washed away after the stimulation. Cell edges are indicated in white. (**b**) Quantification (mean ± S.E.M, n=27/11) based on images like those shown in **a**. Note that dynasore almost completely blocked TSH/TSH receptor internalization in primary mouse thyroid cells. Differences are statistically significant by two-way ANOVA. ***, $P < 0.001$ by Bonferroni´s post hoc test**.**

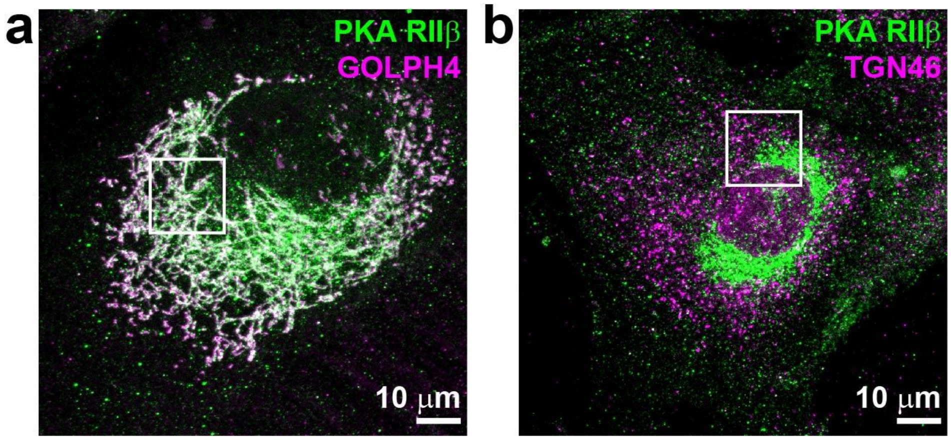

Supplementary Figure 7: Confocal image stacks of PKA RIIβ colocalization with GOLPH4 (a) and TGN46 (b). Shown are z-projections of the image stacks. The regions corresponding to the white boxes in **a** and **b** were used to generate the 3D images shown in **Figure 4b** and **c,** respectively. Data are representative of 3 independent experiments.

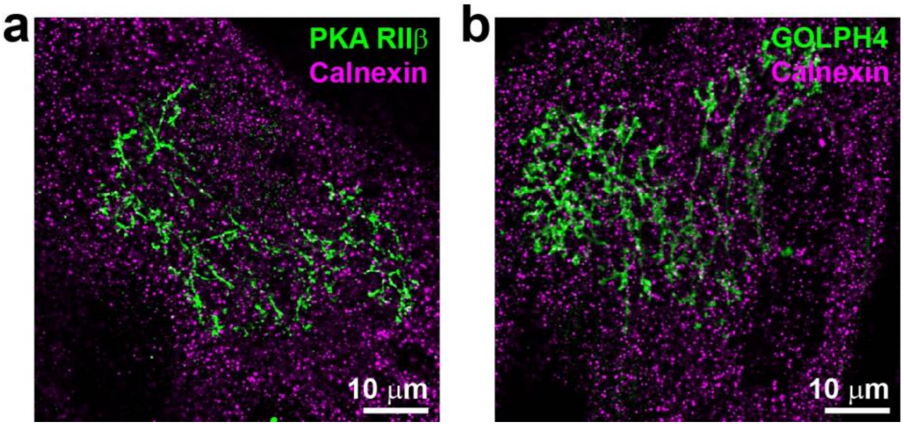

Supplementary Figure 8: Double immunofluorescent staining for RIIβ (**a**) or GOLPH4 (**b**) and the ER marker calnexin. Data are representative of 3 independent experiments.

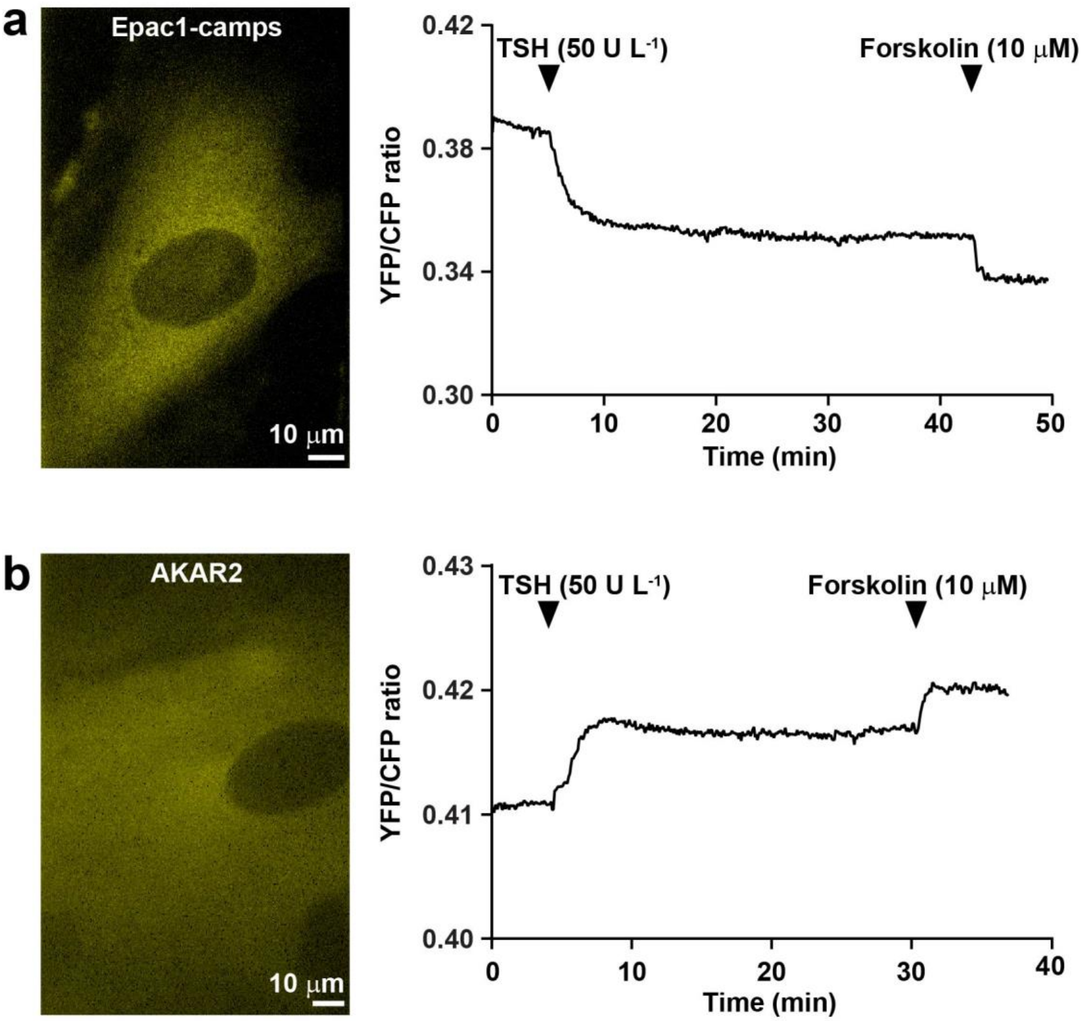

Supplementary Figure 9: Raw traces of cAMP and PKA FRET measurements. (**a**) Left, YFP image of a primary mouse thyroid cell isolated from a transgenic mouse expressing the FRET-based cAMP sensor Epac1-camps. Right, representative cAMP FRET response to TSH stimulation. Note that, since the FRET signals obtained with the Epac1-camps sensor are inversely related to the intracellular cAMP levels, YFP/CFP ratios were subsequently inverted for better comparison with PKA FRET data. (**b**) Left, YFP image of a primary mouse thyroid cell transfected with the FRET-based PKA sensor AKAR2. Right, representative PKA FRET response. At the end of each experiment, cells were stimulated with the direct adenylyl cyclase activator forskolin for normalization.

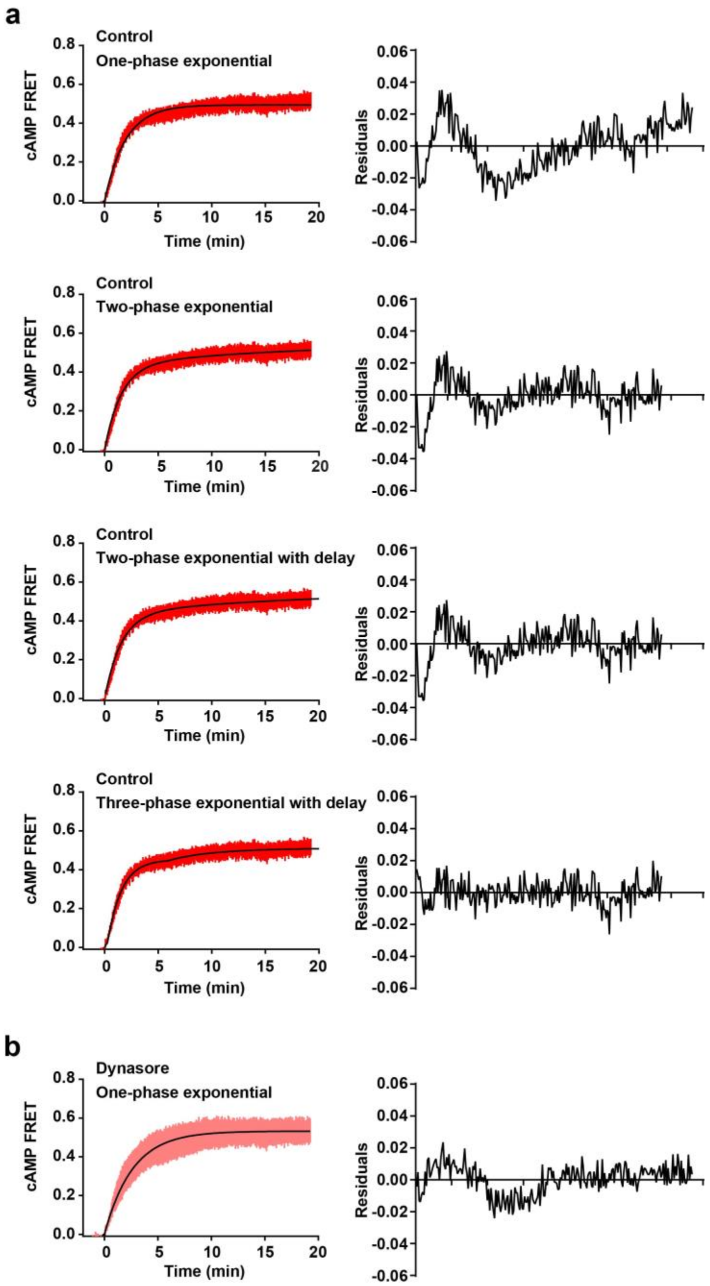

Supplementary Figure 10: Comparison of different models for fitting of cAMP FRET responses to TSH stimulation. The cAMP FRET traces obtained from primary mouse thyroid cells isolated from transgenic Epac1-camps (**Fig. 5a)** were fitted using different models, as indicated. Note that the data obtained without dynasore (**a**) were better fitted with a three-phase exponential model with delay. In contrast, the one obtained with dynasore (**b**) could be already better fitted with a simpler (i.e. one-phase exponential) model.

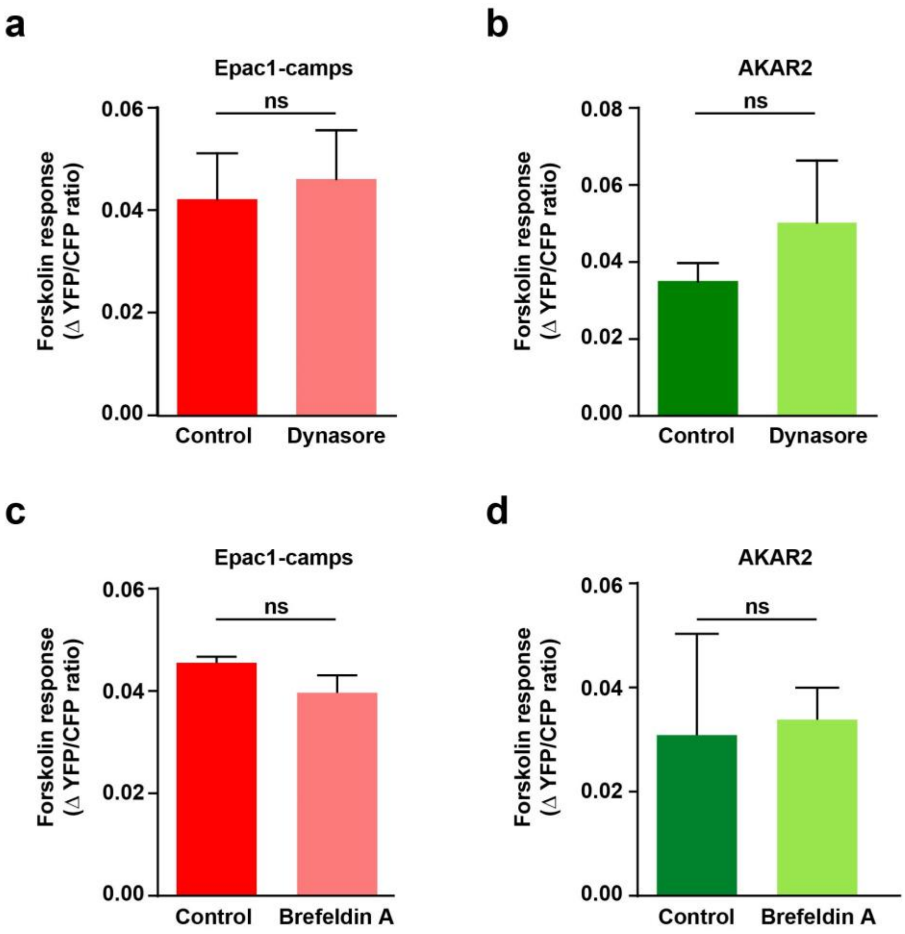

Supplementary Figure 11: Dynasore and BFA treatment do not affect forskolin-induced cAMP and PKA responses. Primary mouse thyroid cells isolated from Epac1-camps transgenic mice (**a,c**) or transected with the global AKAR2 sensor (**b,d**) were preincubated with either dynasore (80 μM) (**a,b**) or BFA (10 μg ml^{-1}) (**c,d**) for 30 min, followed by stimulation with forskolin (10 μM). Shown are the absolute changes of FRET ratio (mean ± S.E.M) compared to control. n=15/11 (**a**), 18/15 (**b**), 14/11 (**c**) and 11/11 (**d**). ns, statistically non-significant difference by Student's t-test.

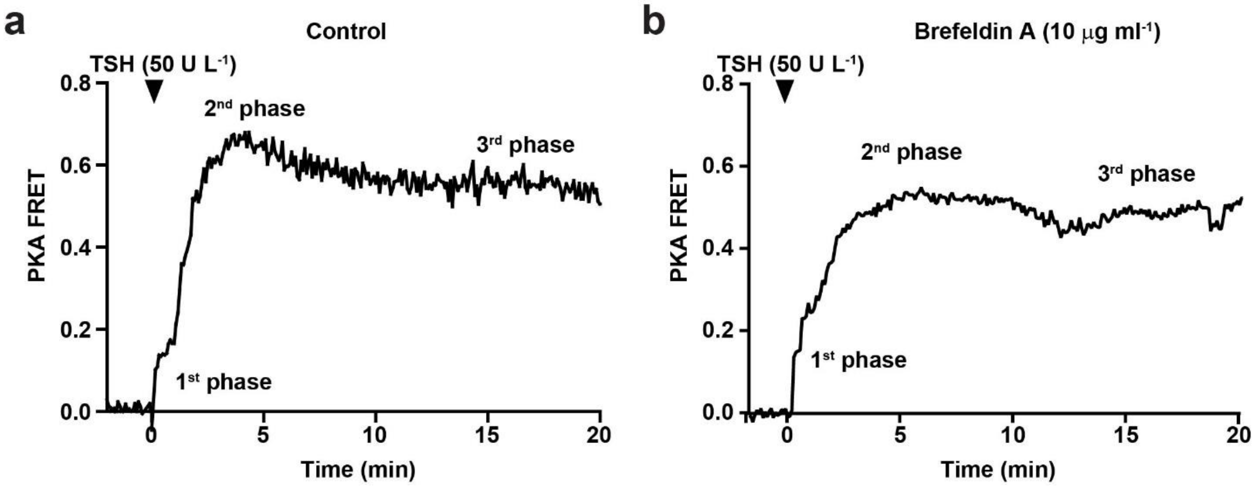

Supplementary Figure 12: Individual PKA FRET traces. Primary mouse thyroid cells were transfected with the FRET-based PKA sensor AKAR2. (**a**) Representative individual trace of PKA response to stimulation with TSH. (**b**) Representative individual trace of PKA response to TSH in the presence of BFA.

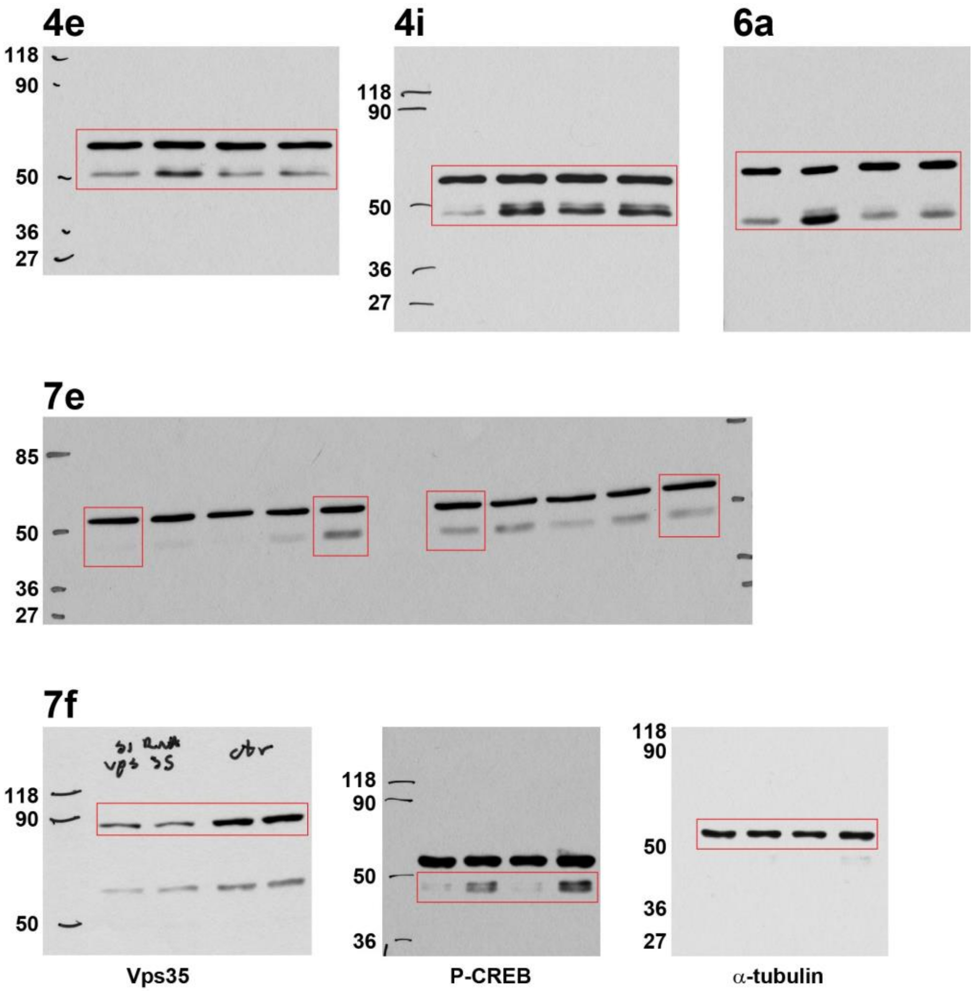

Supplementary Figure 13: Uncropped scans of immunoblots. Marked area in red boxes refer to the cropped images in corresponding Figures as indicated on the top left corner. Protein molecular weight markers (in kDa) are indicated on the left.

Supplementary Table 1: Fitting parameters for the curves in Figure 5b.

	1st phase		2nd phase			3rd phase		
	**Amplitude (A_1)**	**τ_1 (min)**	**Amplitude (A_2)**	**τ_2 (min)**	**Delay (Δt_2)**	**Amplitude (A_3)**	**τ_3 (min)**	**Delay (Δt_3)**
**Control**	0.17 ± 0.05	0.25 ± 0.12	0.36 ± 0.04	0.93 ± 0.16	0.79 ± 0.21	0.02 ± 0.02	0.56 ± 0.46	6.69 ± 1.56
**Dynasore**	0.16 ± 0.01	0.22 ± 0.08	0.02 ± 0.02	2.37 ± 1.77	3.88 ± 3.10	0.09 ± 0.63	10.72 ± 9.67	13.00 ± 3.52

Data were fitted with the equation reported in Methods.
